# Three-Dimensional Real-Time Multiplanar Reconstruction for Intraprocedural Guidance of Challenging Mitral Transcatheter Edge-to-Edge Repair

**DOI:** 10.1016/j.case.2025.02.007

**Published:** 2025-04-18

**Authors:** Krystle Lander, Khin May Thaw, Dale Murdoch, Katherine Lau, Christopher Raffel, Darren L. Walters, Gregory Scalia

**Affiliations:** a Department of Cardiology, The Prince Charles Hospital, Chermside, Australia; b Faculty of Health, Medicine and Behavioural Sciences, University of Queensland, Herston, Australia; c School of Mechanical, Medical and Process Engineering, Faculty Science and Education University of Technology, Brisbane, Australia

**Keywords:** Mitral regurgitation, Mitral transcatheter edge-to-edge repair, 3D multiplanar reconstruction, Real-time multiplanar reconstruction, Structural echocardiology

## Abstract

• 3D MPR allows simultaneous viewing and manipulation of intersecting 2D planes.
• Mitral TEER demands intuitive anatomic displays and optimal imaging precision.
• 3D RT-MPR simplifies complex multidevice and unfavorable anatomy cases.
• Lower resolution in the 3D-derived 2D planar images is a current limitation.

## Graphical abstract

**Figure:**
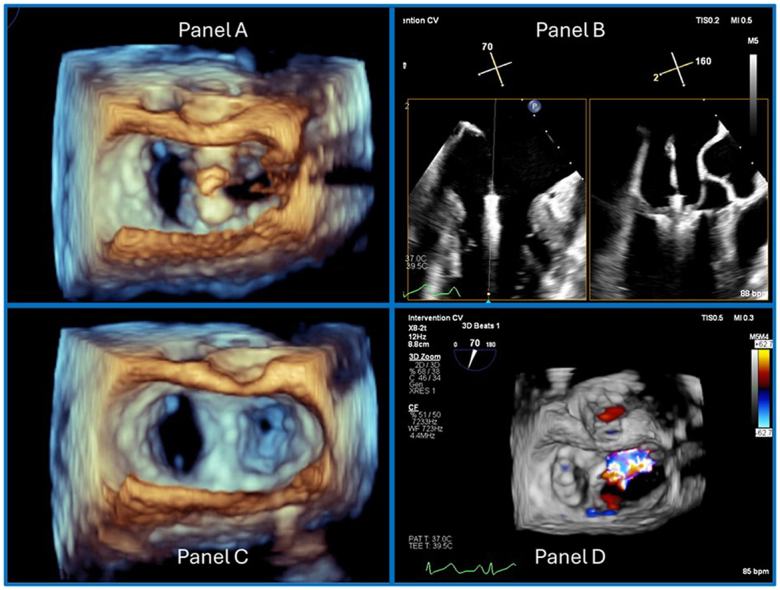

## Introduction

The use of the mitral transcatheter edge-to-edge repair (M-TEER) for treating patients with mitral regurgitation (MR) has grown significantly over the past decade. However, the complex anatomy of the mitral valve (MV) can present challenges during the procedure, particularly when dealing with unfavorable anatomic features such as annular dilation or complex leaflet shapes. In such cases, traditional two-dimensional (2D) and three-dimensional (3D) transesophageal echocardiography (TEE) may not provide the detailed imaging required for precise device placement.

Real-time 3D multiplanar reconstruction (RT-MPR; with vendor-specific nomenclature, e.g., Philips’s Live MPR and GE’s Flexi-slice) demonstrates significant promise in improving M-TEER procedures by allowing clinicians to simultaneously view multiple 2D planes and 3D volumes. This approach is particularly beneficial for challenging cases with complex valve anatomy, commissural MR, leaflet calcification, and the need for multiple devices.

## Case Presentation

**Figure 1:**
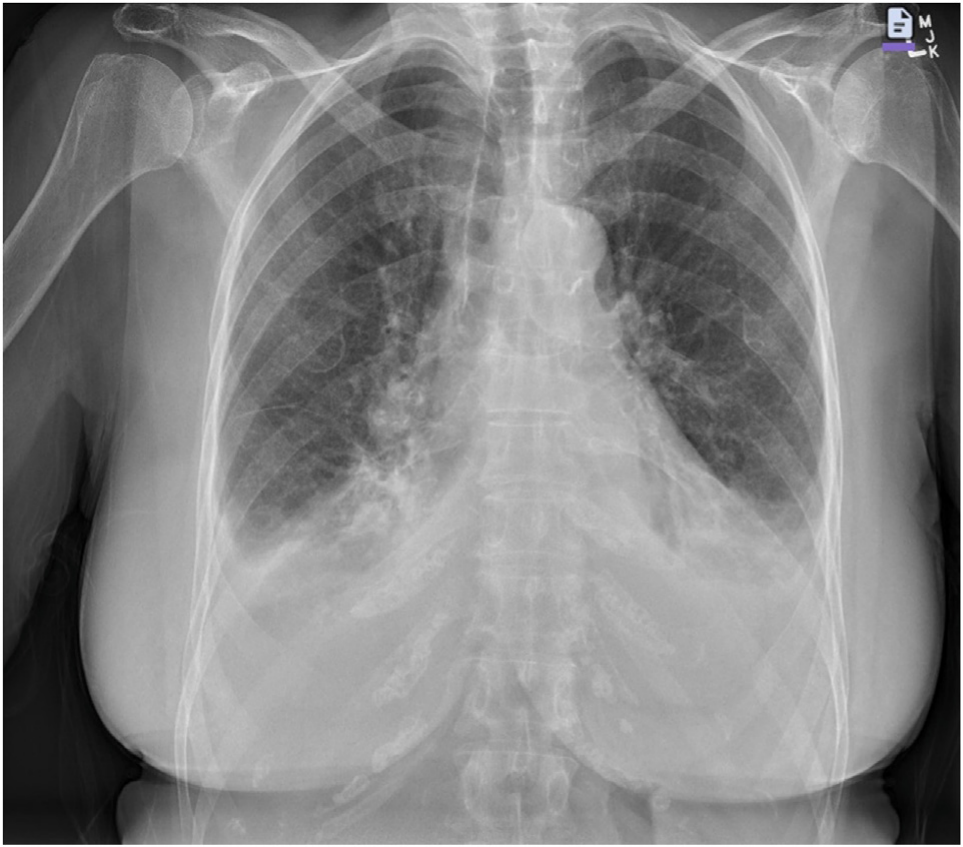
Anteroposterior chest radiography demonstrates pulmonary congestion and bilateral pleural effusions.

An 82-year-old woman presented with decompensated heart failure with clinical and radiologic evidence of pulmonary edema (Figure 1). Clinical examination was suggestive of severe MR, which was confirmed on transthoracic echocardiography.

**Figure 2:**
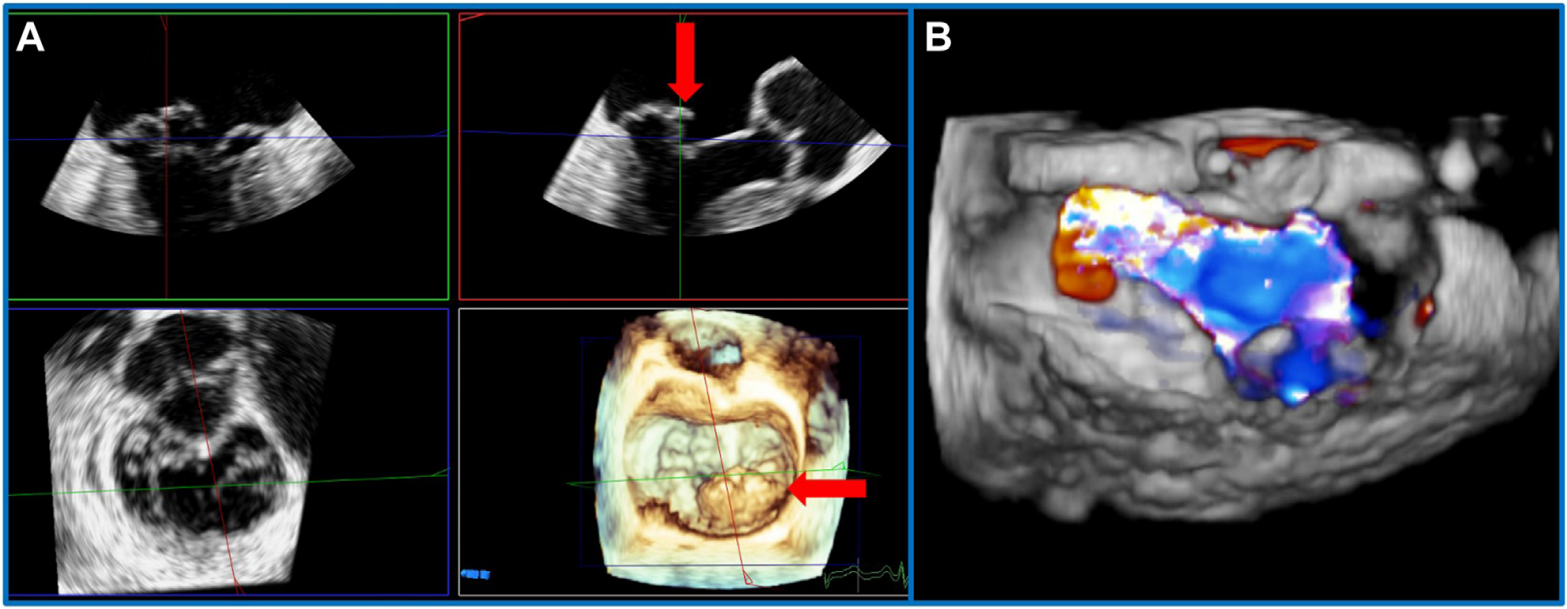
**(A)** Three-dimensional TEE, midesophageal, RT-MPR simultaneous orthogonal long-axis *(top)* and short-axis *(bottom)* 2D and volume-rendered reconstruction en face MV views, demonstrates extensive P_2_/P_3_ prolapse and isolated flail segment *(arrows)*. **(B)** Three-dimensional TEE, midesophageal, volume-rendered reconstruction en face MV view with color flow Doppler, demonstrates severe anteriorly directed MR.

Over the following 7 days, diuresis was effective. However, symptoms of orthopnea and paroxysmal nocturnal dyspnea persisted. TEE was performed, demonstrating extensive prolapse of P_2_/P_3_ with an isolated flail chord and associated severe MR (Figure 2, Video 1, Video 2). Proximal isovelocity surface area–derived effective orifice area was 94 mm^2^, proximal isovelocity surface area regurgitant volume 100 mL, and 3D vena contracta area 120 mm^2^. Three-dimensional MV area (MVA) was 4.9 cm^2^, with a mean pressure gradient (MPG) of 3 mm Hg. A decision was made to undertake inpatient mitral intervention. Given the patient’s age, frailty, and multiorgan dysfunction, plus suitable anatomy on TEE, the heart team decided that M-TEER was the most appropriate treatment. Given the anatomic complexity of the leaflet abnormalities, urgent M-TEER was anticipated to require a two-device strategy.

**Figure 3:**
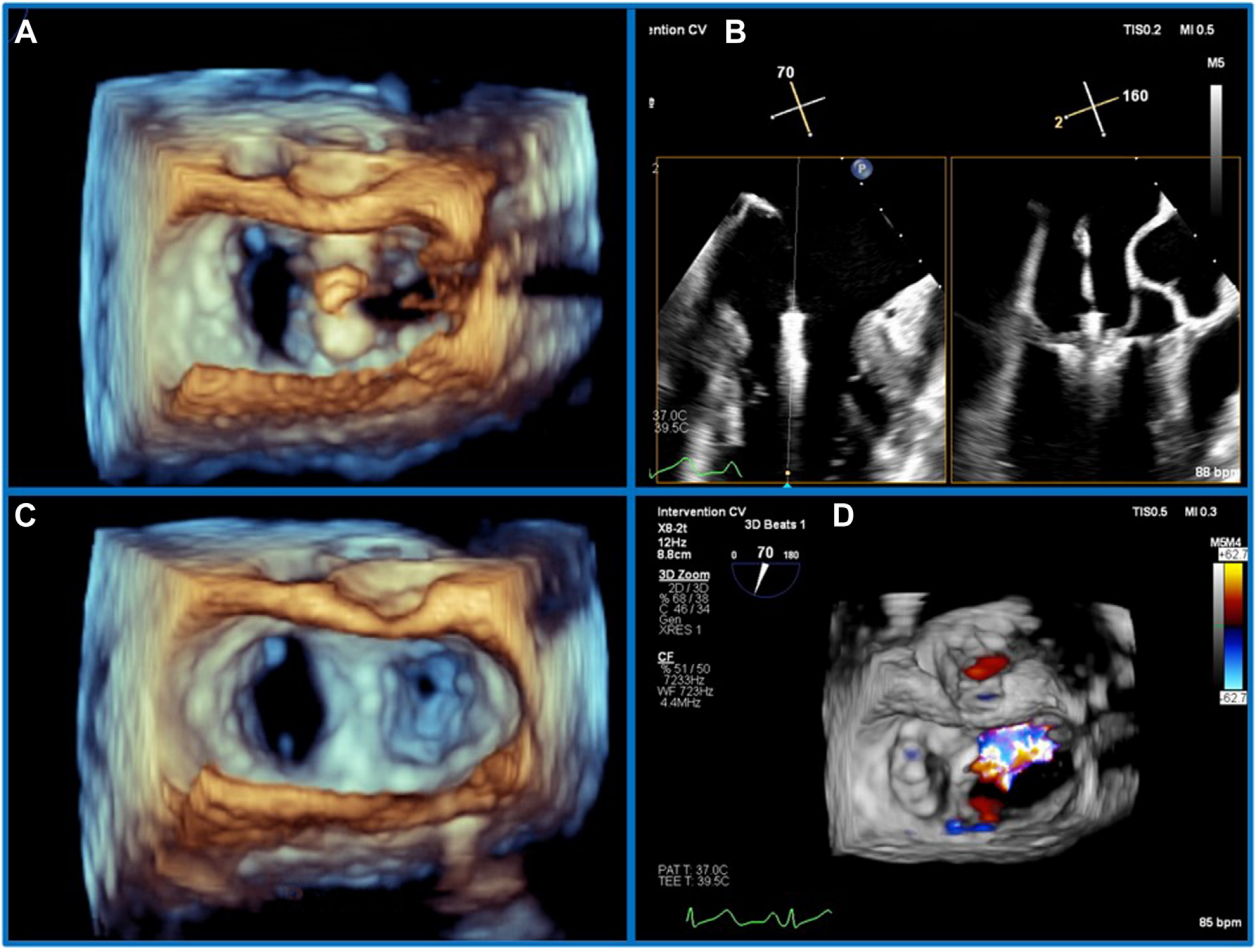
Insertion of first M-TEER device. Three-dimensional TEE, midesophageal, volume-rendered reconstruction en face MV view, demonstrates optimal orientation of the device over central A_2_P_2_**(A)** and confirms a broad tissue bridge after release **(C)**. **(B)** Two-dimensional TEE, simultaneous long-axis display from the bicommissural view, demonstrates optimal grasping view with ideal grasping position of the device. **(D)** Three-dimensional TEE, midesophageal, volume-rendered reconstruction en face MV view with color flow Doppler, demonstrates residual severe MR medial to the device.

**Figure 4:**
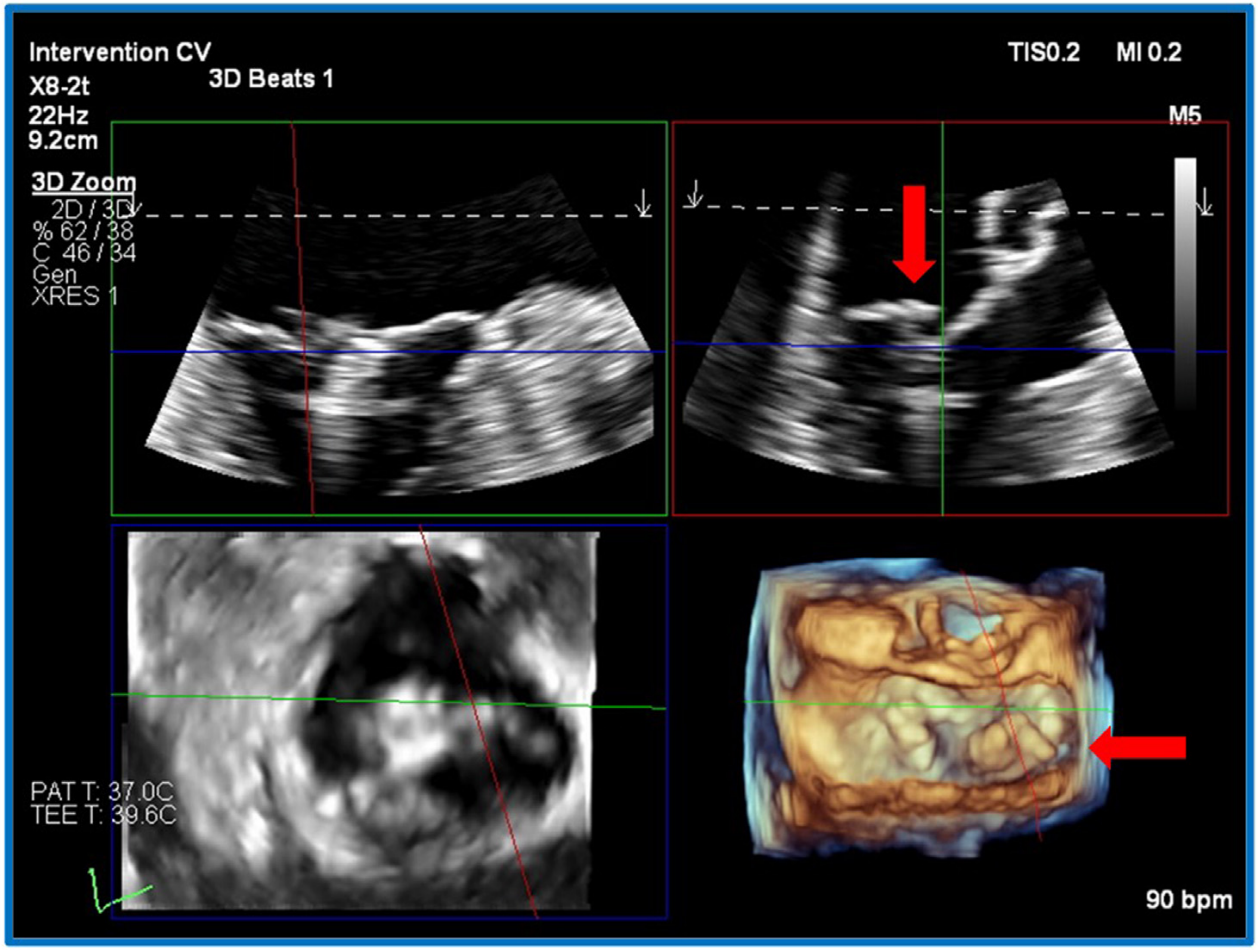
Three-dimensional TEE, midesophageal, RT-MPR simultaneous orthogonal long-axis *(top)* and short-axis *(bottom)* 2D and volume-rendered reconstruction en face MV systolic views after deployment of the first device, demonstrates the detailed valve anatomy at the specific point of the residual MR (*red and green planes* aligned next to the initial device) and confirms the flail segment *(arrows)* medial to the device.

The first device (MitraClip G4 XTW; Abbott Laboratories) was easily placed at the prolapsed segment in central A_2_P_2_ (Figure 3, Video 3), with excellent-quality imaging for grasping. There was severe residual MR medial to this first device (Figure 3, Video 4). The mechanism for the residual MR was thought to be the isolated flail segment medial to the device, shown clearly by RT-MPR (Figure 4, Video 5). The MPG remained at 3 mm Hg, with combined 3D MVA of the two dual orifices of 4.2 cm^2^.

**Figure 5:**
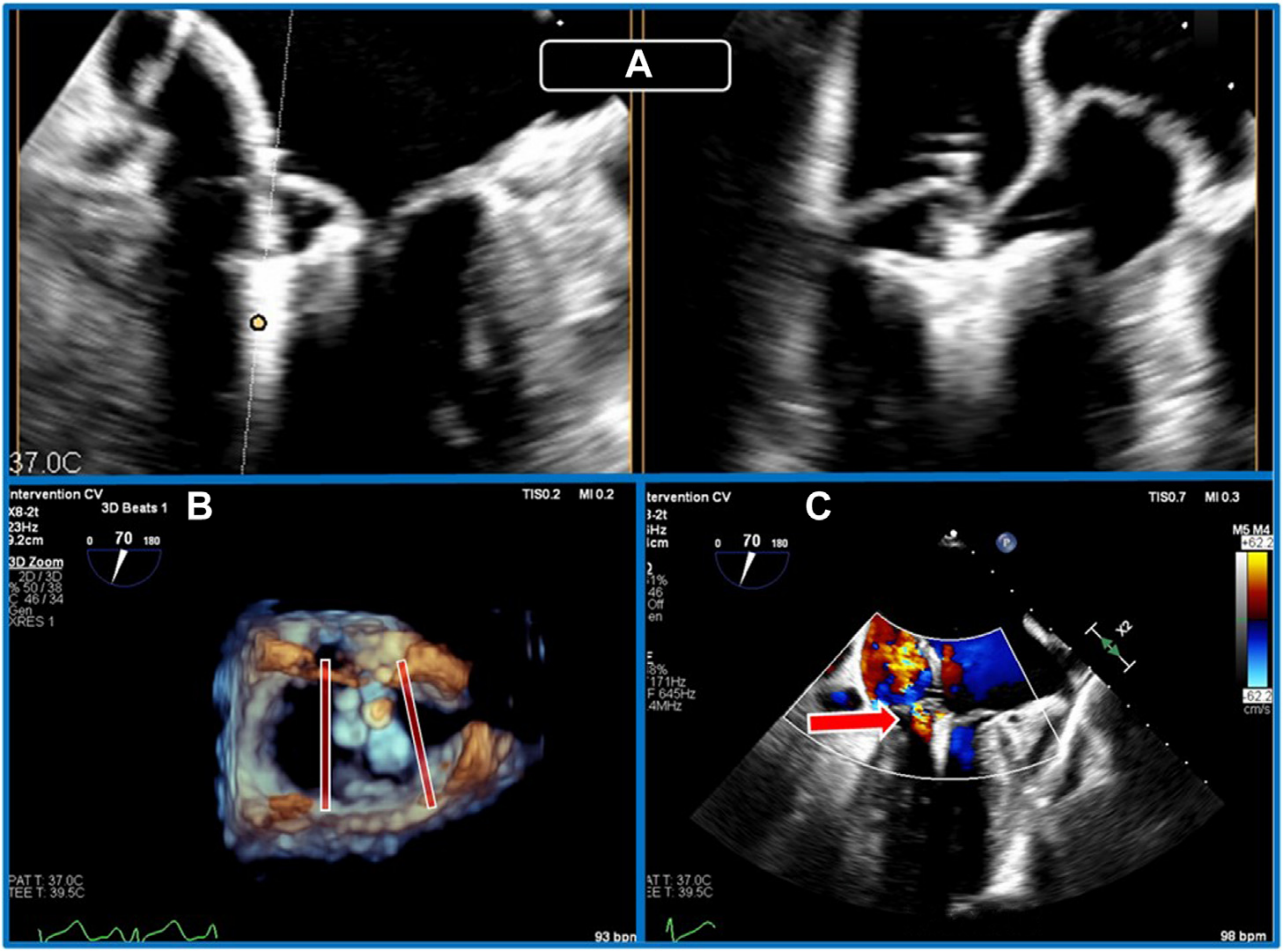
Unsuccessful first attempt at placement of a medial second device. Three-dimensional TEE, midesophageal, RT-MPR simultaneous orthogonal long-axis views **(A)** and short-axis volume-rendered reconstruction en face MV **(B)** and 2D bicommissural (70°) view with color flow Doppler **(C)**, demonstrates optimal grasping view, near parallel alignment of the devices *(red lines)*, and residual MR *(arrow)* despite good grasp (note proximal flow acceleration despite a normal Nyquist limit).

**Figure 6:**
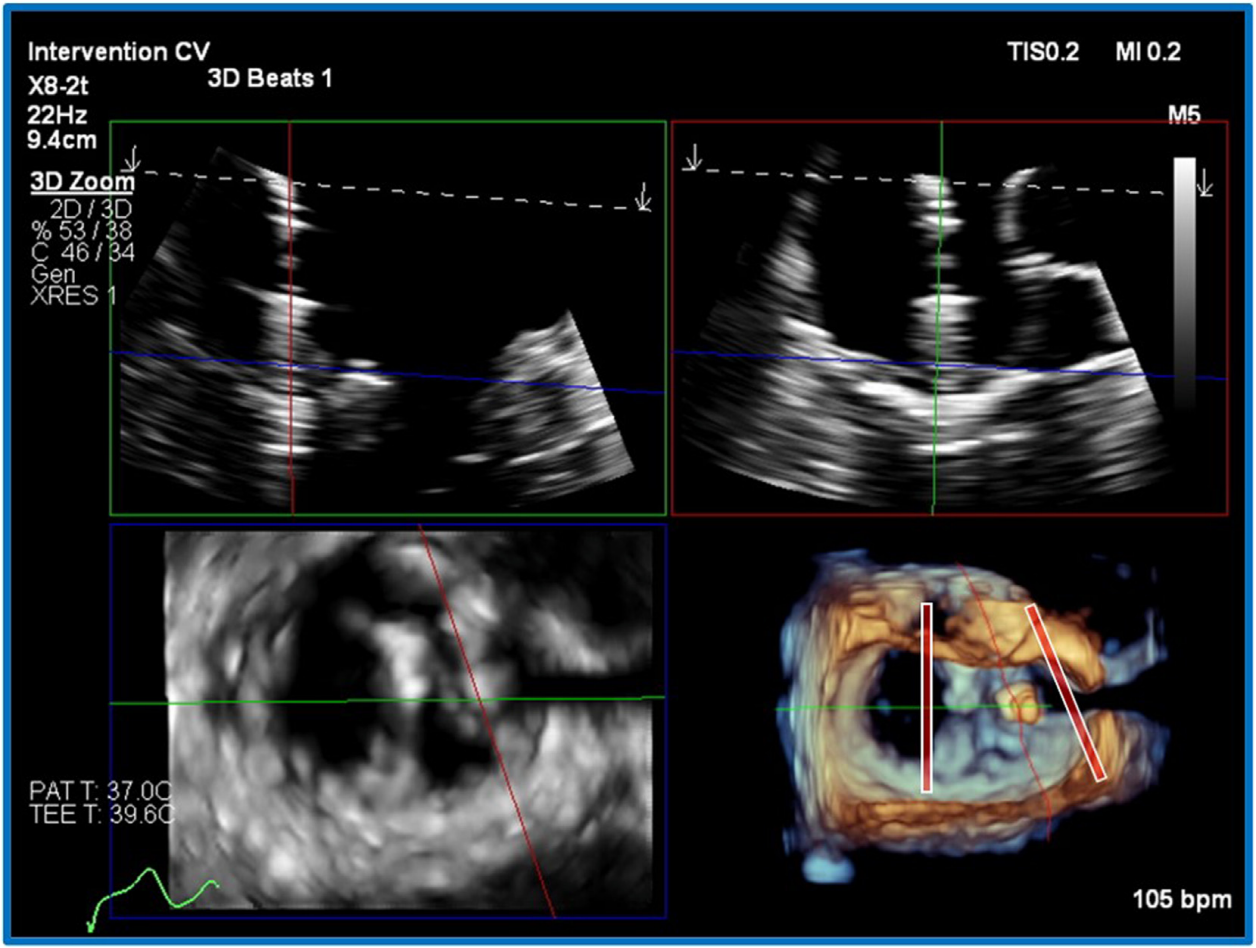
A-frame plan of the second device, second attempt. Three-dimensional TEE, midesophageal, RT-MPR simultaneous orthogonal long-axis *(top)* and short-axis *(bottom)* 2D and volume-rendered reconstruction en face MV views, demonstrate a strategy for simultaneous assessment of an atypical device alignment, while able to better compensate for shadow generated from the first device.

A second device (MitraClip G4 XT) was selected, on the basis of long leaflet length at P_3_, preoperative limited MVA of 4.9 cm^2^, and a postdeployment MPG of 3 mm Hg. Initially, the second device was placed parallel to the first device at the point of flail. This resulted in ongoing significant MR medial to the second device (Figure 5). Subsequently, an “A-frame” strategy was planned, aiming to grasp a more medial aspect of the P_3_ leaflet and central aspect of A_2_ (Figure 6, Video 6). The result was excellent, with trace residual regurgitation (Video 7, Video 8); the MPG was 3 mm Hg.

**Figure 7:**
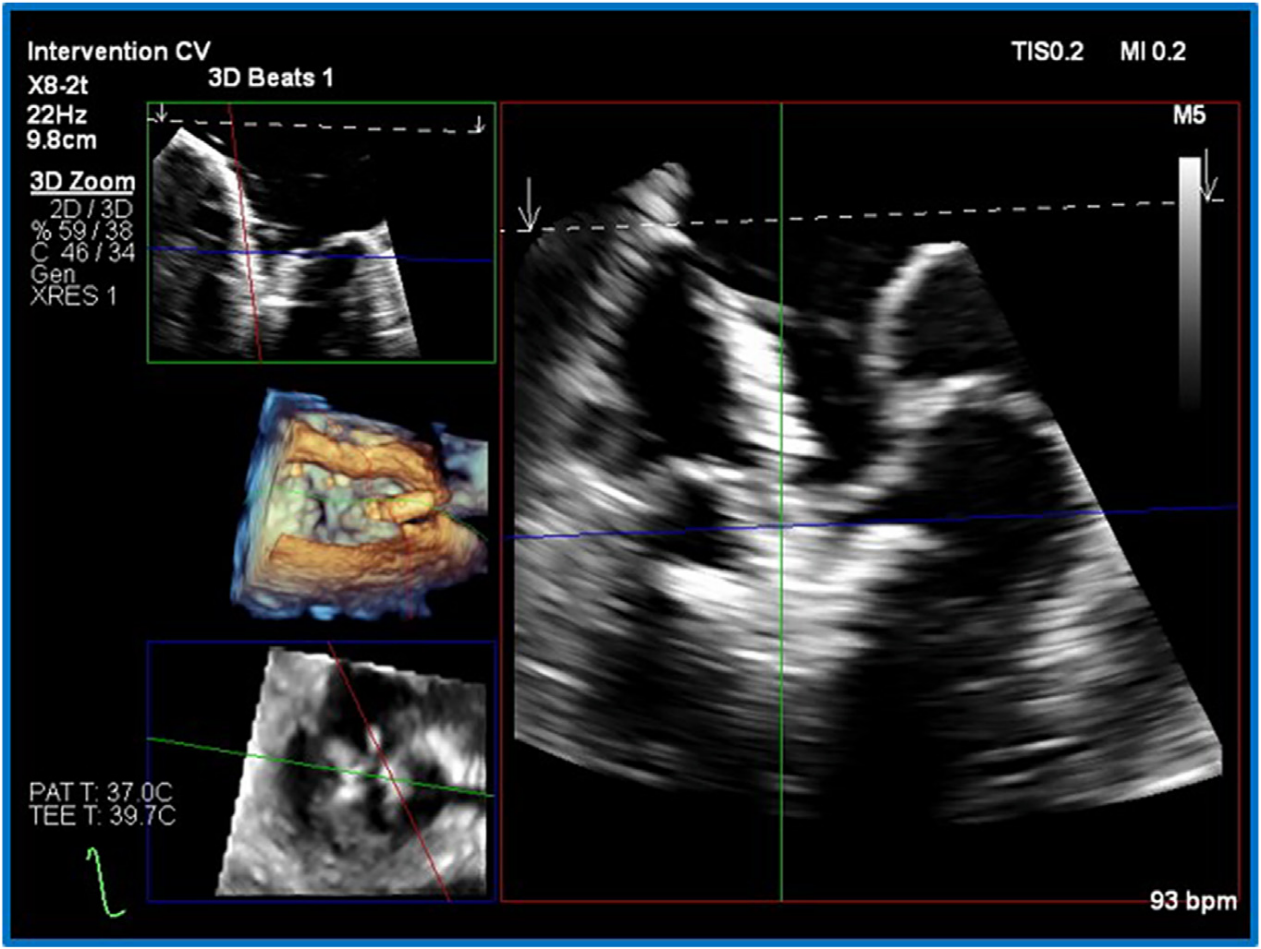
Three-dimensional TEE, midesophageal, RT-MPR simultaneous orthogonal long-axis and short-axis, 2D and volume-rendered reconstruction en face MV views after deployment of the final device, demonstrates how the smaller views on the *left* can be enlarged as necessary to enhance visualization (note that the *red plane* is enlarged on the *right* and confirms adequate leaflet grasping).

RT-MPR provided simultaneous imaging of device orientation (3D anatomic image, Figure 6D, Video 6), leaflet insertion, and device trajectory (Figure 6A and B, Video 6). Images are derived from a 3D zoom block, optimized for frame rate and pixel density. This is particularly helpful when a second device is used, as the location is rarely the simpler central A_2_P_2_ placement. Additionally, shadows are generated by the first device, the guide catheter introducer, and the second device itself (significant shadows are shown in Figure 3D and Video 4). There may only be a narrow ideal window in which to image. Furthermore, the display of RT-MPR can be dynamically altered to highlight the key information required (Figure 7 compared with Figure 4, Figure 5, Figure 6). We have found that the arrangement of Figure 7 is best for the grasping step.

## Discussion

The use of M-TEER for MR has become more frequent, particularly with US Food and Drug Administration approval for both primary and secondary MR.^1^, ^2^, ^3^, ^4^ Unfavorable anatomic features, such as significant annular dilation, short leaflet lengths, complex leaflet morphology, and noncentral MR jets, can particularly present challenges during the procedure.^3^ Historically, 2D biplane views built from a bicommissural base image and an orthogonal long-axis “grasping view,” have been used to confirm optimal position and orientation of the device.^4^^,^^5^ However, in complex cases, these standard 2D and 3D transesophageal echocardiographic imaging techniques may not provide adequate anatomic spatial anatomic visualization required for optimal device placement.

The advent of RT-MPR has enabled simultaneous cross-referencing of multiple high-resolution 2D planes within a comprehensive 3D volumetric representation. By displaying the data along different planes, such as axial, sagittal, or coronal, clinicians can obtain various views that help with diagnosis and planning treatments. The ability to rotate around a common axis allows further exploration of the data, providing a comprehensive understanding of the anatomy or pathology involved.^6^^,^^7^ In the cardiac interventional catheterization laboratory, this imaging revolution is progressively enhancing accuracy, efficiency of analysis, and procedural success, often in cases previously thought to be technically challenging.

During M-TEER, RT-MPR allows more precise manipulation of the required 2D projections, optimizing device interaction with the MV, annulus, and leaflets. This ensures that the device is positioned directly at the problematic segment and is aligned perpendicular to the line of coaptation. This enhanced spatial awareness for the operators also reduces the risk for complications such as device misplacement, leaflet damage, or improper leaflet capture. This technique is of particular benefit when placing multiple devices, minimizing potential risks such as interference with chordae tendineae or damage to the valve apparatus.^6^^,^^7^

Aside from the technical advantages noted above, RT-MPR may offer some safety advantages over traditional imaging. Esophageal bleeding and perforation are associated with prolonged procedure time, and increased manipulation.^8^, ^9^, ^10^ By providing a more comprehensive view of the MV and surrounding structures, RT-MPR may reduce the procedure time and the need for multiple probe adjustments.^10^^,^^11^ However, RT-MPR has some limitations, with the temporal and spatial resolution of the 3D-derived 2D planar images being significantly lower than equivalent native 2D-derived biplane imaging. Visually, the screen layout by default consists of four small boxes (see Figures 4 and 6), each notably smaller than a standard 2D image. The use of alternative layouts (see Figure 7) can help overcome this. Training echocardiographers to generate 3D volume data and then construct 2D planar images has a steep learning curve. As RT-MPR may involve nonstandard views and techniques, it is essential that all team members become familiar with the modality to ensure effective collaboration and optimal procedural outcomes.^7^^,^^10^^,^^12^ It should be remembered that the net goal is to achieve useful, interpretable, and intuitive images for all members of the interventional team.

## Conclusion

The transition from traditional 2D TEE to 3D TEE with RT-MPR represents a major advance in structural cardiac imaging. By enhancing visualization of MV anatomy, RT-MPR allows more accurate anatomic assessment and device manipulation, potentially minimizing complications and improving procedural outcomes, particularly in complex cases. Although there is a substantial learning curve and some technical limitations, the incremental value of RT-MPR will enable progressively more complex M-TEER procedures, with enhanced procedural success and optimized clinical outcomes for patients.

## Ethics Statement

The authors declare that the work described has been carried out in accordance with The Code of Ethics of the World Medical Association (Declaration of Helsinki) for experiments involving humans.

## Consent Statement

Complete written informed consent was obtained from the patient (or appropriate parent, guardian, or power of attorney) for the publication of this study and accompanying images.

## Funding Statement

The authors declare that this report did not receive any specific grant from funding agencies in the public, commercial, or not-for-profit sectors.

## Disclosure Statement

The authors report no conflict of interest.

## Supplementary Data

Video 1 Three-dimensional TEE, midesophageal, volume-rendered reconstruction en face MV view, demonstrates extensive P_2_/P_3_ prolapse and isolated flail segment.

Video 2 Three-dimensional TEE, midesophageal, volume-rendered reconstruction en face MV view with color flow Doppler, demonstrates extensive P_2_/P_3_ prolapse, isolated flail segment, and severe, anteriorly directed MR.

Video 3 Two-dimensional TEE, simultaneous long-axis display from the bicommissural view, demonstrates optimal grasping view with ideal grasping position of the device.

Video 4 Three-dimensional TEE, midesophageal, volume-rendered reconstruction en face MV view with color flow Doppler after deployment of the first device, demonstrates residual severe MR medial to the device.

Video 5 Three-dimensional TEE, midesophageal, RT-MPR simultaneous orthogonal long-axis *(top)* and short-axis *(bottom)* 2D and volume-rendered reconstruction en face MV views after deployment of the first device, demonstrates the detailed valve anatomy at the specific point of the residual MR (*red and green planes* aligned next to the initial device) and confirms the flail segment medial to the device.

Video 6 A-frame plan of the second device, second attempt. Three-dimensional TEE, midesophageal, RT-MPR simultaneous orthogonal long-axis *(top)* and short-axis *(bottom)* 2D and volume-rendered reconstruction en face MV views demonstrate a strategy for simultaneous assessment of an atypical device alignment while able to better compensate for shadow generated from the first device.

Video 7 Three-dimensional TEE, midesophageal, volume-rendered reconstruction en face MV view after successful deployment of the second device, demonstrates the detailed valve anatomy with devices placed at A_2_P_2_ and medial A_2_P_2_ without a flail segment.

Video 8 Three-dimensional TEE, midesophageal, volume-rendered reconstruction en face MV view with color flow Doppler after successful deployment of the second device, demonstrates trace MR.
